# An improved multiple-locus variable-number of tandem repeat analysis (MLVA) for the fish pathogen *Francisella noatunensis* using capillary electrophoresis

**DOI:** 10.1186/1746-6148-9-252

**Published:** 2013-12-13

**Authors:** Samuel Duodu, Xihe Wan, Nora Martinussen Tandstad, Pär Larsson, Kerstin Myrtennäs, Andreas Sjödin, Mats Forsman, Duncan J Colquhoun

**Affiliations:** 1 Section for Bacteriology, Norwegian Veterinary Institute, Oslo, Norway; 2 Laboratory of Marine biotechnology, Jiangsu Institute of Oceanography and Marin fisheries, Nantong 226007, China; 3 Swedish Defence Research Agency (FOI), CBRN Defence and Security, Umeå, Sweden

**Keywords:** VNTR, Capillary electrophoresis, *Francisella noatunensis*, Fish

## Abstract

**Background:**

Francisellosis, caused by the bacterium *Francisella noatunensis* subsp. *noatunensis*, remains a serious threat to Atlantic cod (*Gadhus morhua*) farming in Norway and potentially in other countries. As outbreak strains appear clonal in population structure, access to highly discriminatory typing tools is critical for understanding the epidemiology of francisellosis infections in aquaculture. In this study, a simplified multiple-locus variable-number of tandem repeat analysis (MLVA) targeting five highly polymorphic variable number of tandem repeat (VNTR) loci in a single multiplex PCR was developed to rapidly discriminate between outbreak strains.

**Results:**

The assay resulted in identification of at least 13 different allelic profiles or subpopulations among 91 *F. noatunensis* isolates from farmed cod in Norway. The VNTR loci appear relatively stable, with isolates originating from individual outbreaks showing identical MLVA profiles following repeated passage. MLVA displayed greater discriminatory power than pulse-field gel electrophoresis (PFGE). Both MLVA and PFGE show good epidemiological concordance by their abilities to separate outbreak strains from epidemiologically unrelated isolates.

**Conclusions:**

The MLVA method presented here is robust, easy to perform and provides a good alternative to other typing systems for *F. noatunensis* subsp. *noatunensis* and epidemiological study of francisellosis in cod.

## Background

*Francisella noatunensis* is an emergent fish pathogen of major concern. It causes francisellosis, a systemic bacterial disease characterized by the presence of multi-organ granuloma with high morbidity and varying associated mortality levels [1]. The disease affects several important cultured fish species in fresh, brackish and marine water environments. Francisellosis in farmed Atlantic cod *Gadhus morhua* L. was first reported in 2004/2005, when an outbreak caused by *F. noatunensis* subsp. *noatunensis* was discovered in Norway [2,3]. Since then, many cases of *F. noatunensis* infection have been diagnosed in cod from numerous grow-out facilities spanning most of the mid-and south-western Norwegian coastline. Outbreaks caused by different strains of *F. noatunensis* have also been linked to other important cultured fish species around the globe [1]. As no vaccine is yet available and antibiotic treatment largely ineffective, understanding the infection dynamics and spread of the disease may be important in regard to management of francisellosis in aquaculture.

Genetically, *F. noatunensis* subsp. *noatunensis* is a monomorphic pathogen, showing very little intra-species variation [4,5]. As a result, few genetic tools with the necessary resolution to track and link individual disease outbreaks are available. An increasingly applied molecular typing tool for discrimination of bacterial species with stable clonal population structures is multiple locus variable-number tandem repeat analysis (MLVA). The method is based upon PCR amplification of variable tandem repeats (VNTRs), which are short polymorphic DNA sequences located at several loci in many microbial genomes [6]. Isolates belonging to single bacterial species generally maintain the same sequence elements but show variation in the number of repeat units through the activity of a strand-slippage mechanism introduced by DNA polymerase during replication [7-9]. The different variants are most commonly resolved by standard agarose gel electrophoresis or capillary electrophoresis on a DNA sequencer [10].

To date, there is limited information on MLVA application relating to pathogenic fish *Francisella* species. In a recently published study, DNA sequencing was used to determine variations in the number of repeats at seven VNTR loci [11]. Although the study highlighted the value of using MLVA as a typing system for *F. noatunensis* subsp. *noatunensis*, DNA sequencing is costly and labour intensive. Typing of a single isolate may require seven different PCRs and 14 sequencing reactions. The primary aim of the present study was, therefore, to improve the efficiency of MLVA as a typing scheme for discrimination of *F. noatunensis* isolates, through development of a single tube multiplex PCR amplification followed by automated fragment analysis using capillary electrophoresis. The epidemiological utility of the developed assay was compared with Pulsed-field gel electrophoresis (PFGE), which has been the gold standard method for epidemiological investigation of disease outbreaks in many pathogenic bacteria.

## Results

### *MLVA*

**Figure 1:**
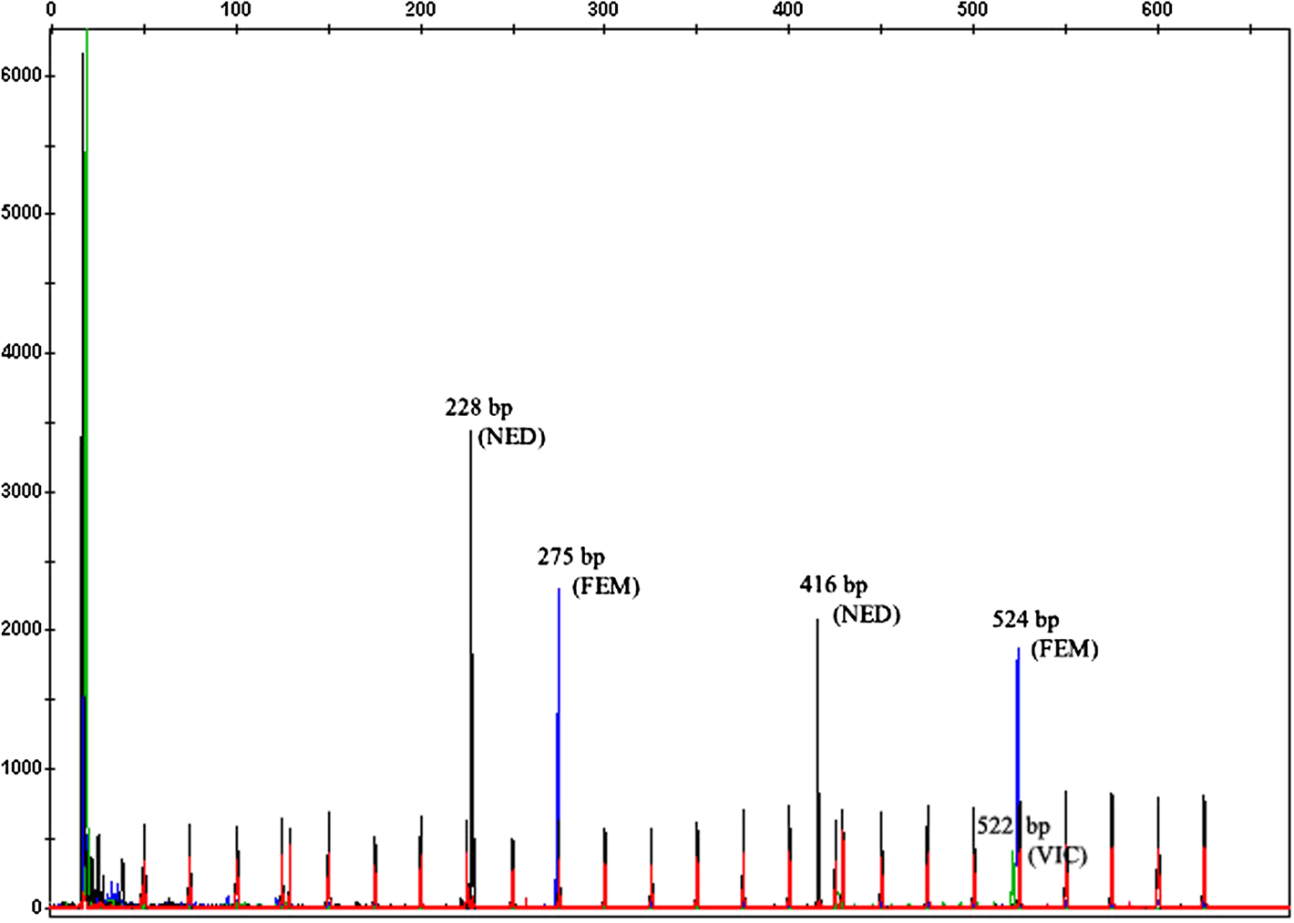
**Electropherograms showing PCR fragments of all five VNTR loci co-amplified in a single PCR reaction.** The fragments were resolved by size and dye colour using capillary electrophoresis.

The MLVA assay developed in this study is based on capillary electrophoresis of the five most polymorphic loci identified within available fish-pathogenic *Francisella* genomes. Four of these loci, fnVNTR-2, fnVNTR-3, fnVNTR-4 and fnVNTR-5 were independently identified as Fnn-VNTR2, Fnn-VNTR3, Fnn-VNTR1 and Fnn-VNTR4, respectively, in a previous study by Brevik et al. [11]. The performance of the present assay was optimised in a single multiplex by determining the appropriate concentrations of primers, PCR amplification and capillary run conditions. Primers chosen for each marker yielded PCR products compatible with the internal size standard which generated peaks from 50 to 625 bp. DNA extracted by boiling was found adequate for further analysis, thus a separate DNA purification step was considered unnecessary. Individual PCR amplicons were easily differentiated based on colour, size and peak height in the electropherograms (Figure 1). Most markers appeared as single sharp peaks, while some showed split peaks, which is a common artefact in capillary electrophoresis, due to non-templated (incomplete) 3′A nucleotide additions (Applied Biosystems). Sizing of individual fragments was reproducible across replicated capillary electrophoretic runs. Sequencing of selected isolates (including all variants identified at each locus) confirmed that the size determined by fragment analysis was due to varying numbers of repeats at each targeted loci. However, calculation of the number of repeats in locus fnVNTR-1 based on automated fragment sizing was inaccurate. Capillary electrophoresis consistently estimated fragment sizes larger than direct sequencing of the different alleles, regardless of the dye (FAM or VIC) used for labeling. Nevertheless, the size differences were consistent across the entire size range for this locus.

*In vitro* stability of the VNTR loci was evaluated after multiple subcultures of four *F. noatunensis* subsp. *noatunensis* isolates displaying genetically distinct MLVA profiles. The isolates were passaged at 5 day intervals for nearly 30 weeks on CHAB incubated at 22°C. Screening of single colonies picked from passage 10, 20, 30 and 40 revealed the same MLVA profile as the original isolates (data not shown).

MLVA performed on 91 *F. noatunensis* subsp. *noatunensis* isolates from Atlantic cod, yielded PCR products for all VNTR loci. The number of individual alleles ranged between four (fnVNTR-4) and nine (fnVNTR-1) (Table 1). FnVNTR-1 carried the largest number of repeats (20–33 repeats), while fnVNTR-4 revealed only a limited number of repeat units (4–7 repeats). Differences in diversity indices (DI) were also observed for the 5 VNTR loci. The highest DI was associated with fnVNTR-1 (76.5%) and the lowest with fnVNTR-2 (57.7%). The discriminatory power for the combined set of VNTR markers was 83.3%, indicating the high probability of the MLVA assay separating two isolates from different diagnostic cases.

**Table 1:** **Characteristics of VNTR loci used in MLVA for *F. noatunensis *** **subsp. *noatunensis *** **isolates**

**Marker locus**	**Alternate designation**^**a**^	**GeneBank accession no.**	**Repeat sequence**	**Number of repeats**	**Fragment size range (bp)**	**No. of alleles**	**DI (%)**	**Coding functions**
fnVNTR-1	NA	CP000937	TCTTTATTG	20-33	427-549	9	76.5	TPR repeat protein
fnVNTR-2	Fnn-VNTR2	GU385768	AGTTATT	8-14	275-440	4	57.7	ThiJ/PfpI family protein
fnVNTR-3	Fnn-VNTR3	GU385769	TAGAT	7-11	213-234	5	67.6	UDP-N-acetylglucosamine 2-epimerase
fnVNTR-4	Fnn-VNTR1	GU385767	TTAAGGTA	4-7	507-532	5	65.5	NA
fnVNTR-5	Fnn-VNTR4	GU385770	CCACAA	9-31	404-539	7	65.5	DNA-directed DNA polymerase

^a^From Brevik et al. [11].

NA, not applicable/available.

**Figure 2:**
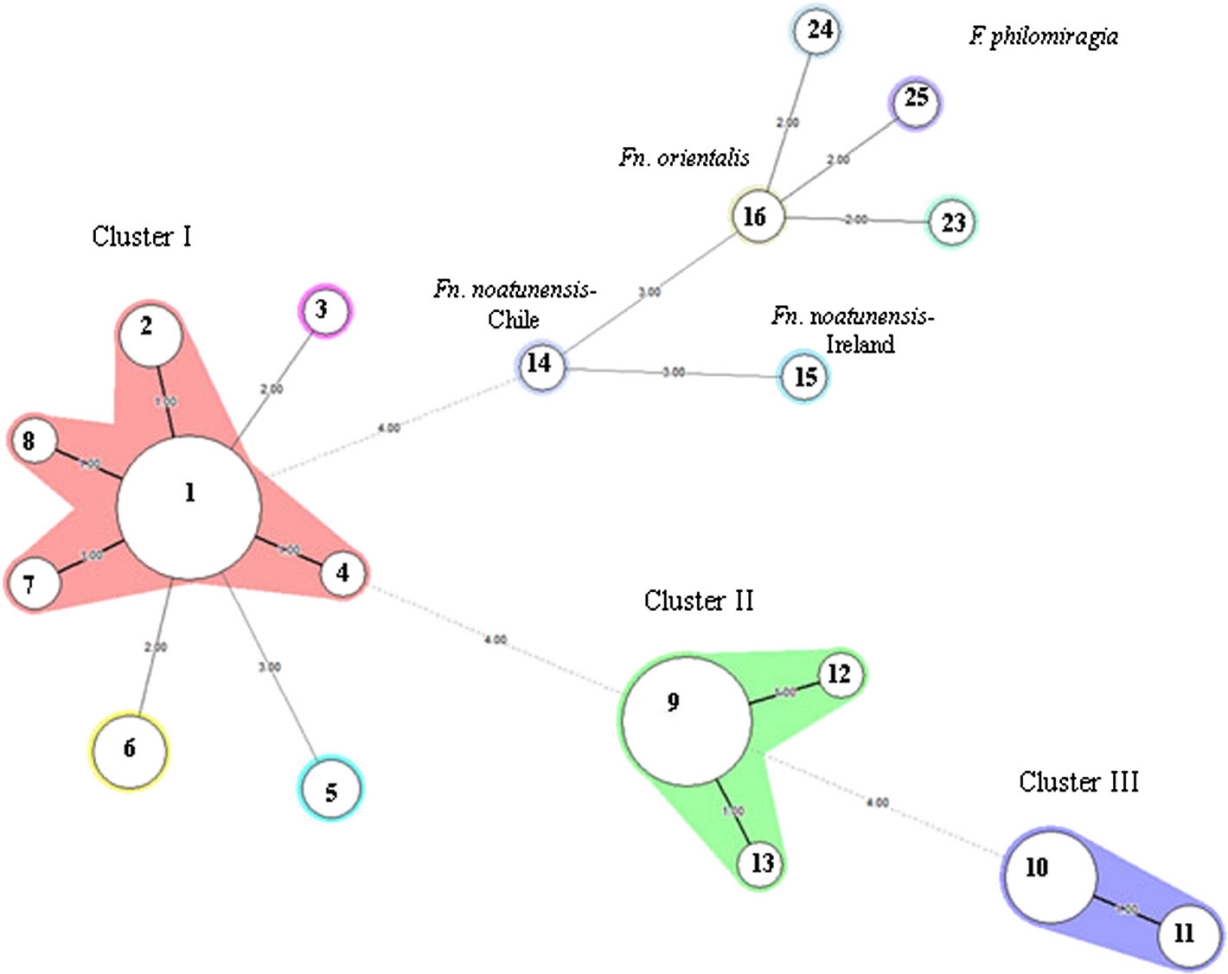
**Minimum spanning tree (MST) of MLVA showing the genetic relatedness among the isolates included in this study.** A categorical coefficient and the priority rule using the highest number of single-locus changes was used for generation of the minimum spanning tree. Each circle in the tree represents a different MLVA type (MVT), with the different MVTs indicated by the number in the circle. MVTs differing by a single VNTR locus are represented by thick short lines, while double-locus variants are shown by thin longer lines and dotted lines for those differing by more than two loci. The number of loci that differ between two MVTs is indicated on the lines linking them together. Clusters were defined as MVTs having a maximum of three differing loci and a minimum cluster size of two. The Norwegian isolates grouped into three main clusters (I, II and III). Cluster I is shaded pink, Cluster II in green and cluster III in violet.

**Figure 3:**
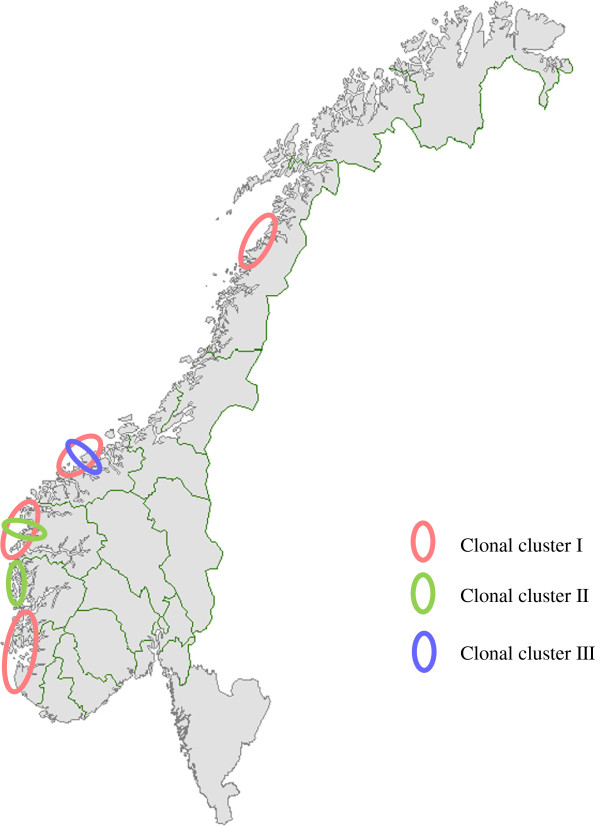
**Map of Norway showing the geographical distribution of *F. noatunensis *subsp. *noatunensis *MLVA clonal groups generated by minimum spanning tree (MST) analysis.** Cluster I is shaded pink, cluster II in green and cluster III in violet.

The population linkage disequilibrium was calculated to be significant by LIAN analysis ($I_{A}^{S}$ = 0.2604, P*para* = 2.21 × 10^-29^), suggesting a low rate of recombination between the alleles. In total, 13 different MLVA types were found among the 91 Norwegian cod isolates (Additional file 1: Figure S1 in supplementary material). These MLVA profiles grouped into three major clonal clusters (designated I, II and III) using minimum spanning tree analysis to display the relationship between the various MLVA types (Figure 2). Cluster I was the largest group containing 8 different MLVA types (types 1, 2, 3, 4, 5, 6, 7 and 8) with MLVA type 1 being the dominant clone. The isolates in this cluster had no specific geographical or temporal associations (Figure 3). Cluster II consisted of 3 different MLVA types (type 9, 12 and 13), with the majority belonging to MLVA type 9 (Figure 2). These isolates were collected from a confined geographical area and included the type strain NCIMB 14265^T^. Cluster III comprised of two closely related MLVA types (types 10 and 11), differing only in one allele. All isolates in this cluster were collected from a limited geographical area in the period of 2006–2008. A large degree of genetic heterogeneity was evident among *F. noatunensis* subsp. *noatunensis* isolates from Chile, Ireland and Norway e.g. the Chilean salmon isolate shared only two VNTR loci with the other *F. noatunensis* subsp. *noatunensis* isolates from cod in Norway (fnVNTR3 and fnVNTR5) and Ireland (fnVNTR2 and fnVNTR3). The few isolates of *F. noatunensis* subsp. *orientalis* and *F. philomiragia* included in this study were distinctly separated from the *F. noatunensis* subsp. *noatunensis* isolates.

### PFGE analysis

**Figure 4:**
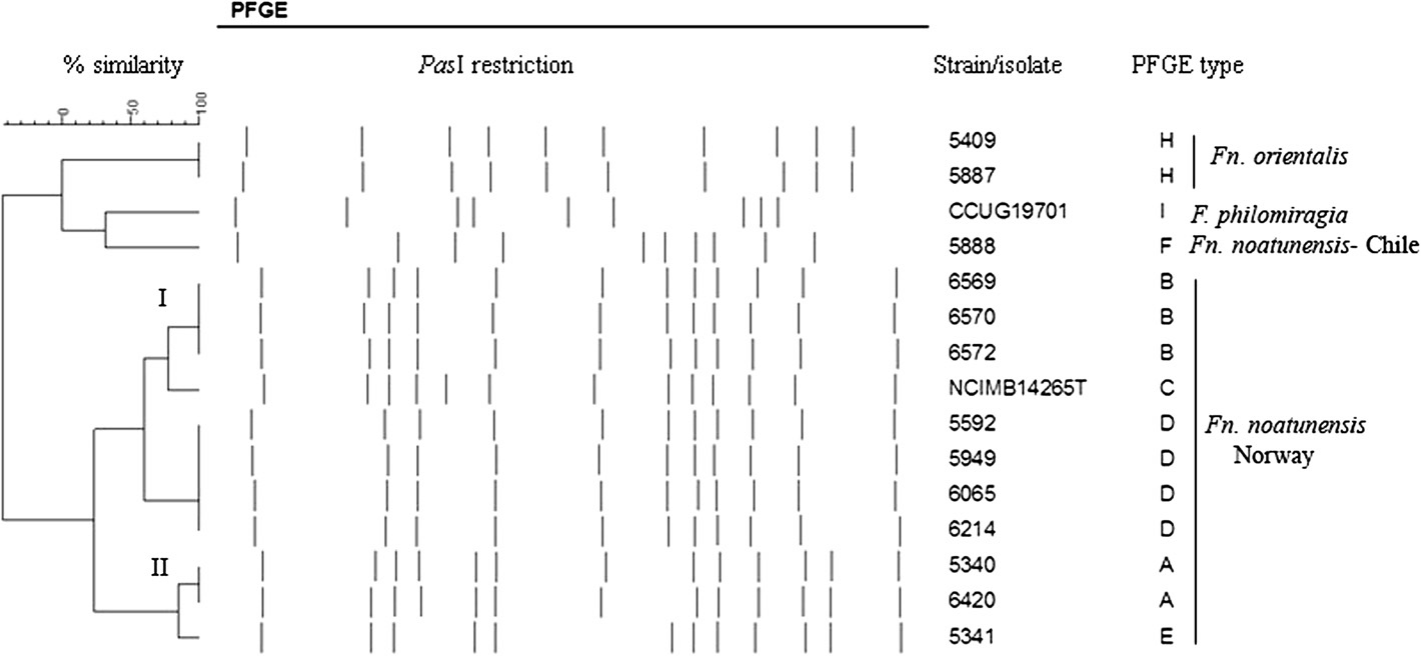
**Dendrogram showing the genetic relatedness among selected isolates based on PFGE analysis.** The dendrogram was constructed using the Dice coefficient correlation and WARD algorithm. The Norwegian isolates grouped into two clusters (I and II).

Overall, PFGE separated the 47 Norwegian cod isolates studied into five PFGE types with a lower discriminatory index (DI = 63.7%; CI 95% = 0.569-0.704) than MLVA typing. The isolates grouped into two main PFGE clusters designated PFGE I and II (Figure 4). The distribution pattern of PFGE types was generally consistent with the clustering obtained for the MLVA typing. In most cases, isolates belonging to the same MLVA cluster belonged to the same PFGE type. Exceptions included isolate 5341 (MLVA cluster I) and NCIMB14265^T^ (MLVA cluster II) that differed in PFGE type from other isolates in their clusters, displaying a single band difference. The non-Norwegian isolates displayed unique PFGE types, which separate distinctly from each other (see Additional file 1: Figure S1 in supplementary material). The concordance between PFGE and MLVA, as determined by the adjusted Rand index, was moderate (50%) for the isolates typed by both methods. The calculated Wallace coefficients, which measures directional congruence between the typing methods were 80.8% and 37.1% for MLVA and PFGE, respectively. This suggests MLVA as more predictive in assigning isolates to the same PFGE group, than vice versa.

## Discussion

In this study we validated a novel MLVA assay for the fish pathogen *F. noatunensis* subsp. *noatunensis* using capillary electrophoresis. The presented assay relies on multiplex PCR amplification of five VNTR loci visualised in a single electrophoretic run, allowing typing of many or few isolates simultaneously. The MLVA protocol is robust, easy to perform and generates unambiguous numerical profiles for individual strain identification.

Not surprisingly, independent searches within highly similar genomes performed during the same time periods identified several common informative VNTR loci. Brevik et al. [11] published an MLVA assay for *F. noatunensis*, in which sequencing was used to identify polymorphisms in seven VNTR loci. Four of these loci were also identified and included in the present study. The fnVNTR-1 marker was, however, unique to the present study. The high degree of polymorphism identified in each VNTR loci suggests that the optimal markers were selected by both MLVA schemes. For all loci, with the exception of fnVNTR-1, accurate size estimation of PCR products was achieved by capillary electrophoresis. Sizing by capillary, although reproducible for this locus, failed to correspond to the actual fragment size identified by sequencing. This discrepancy could be due to the nature of the gel matrix, or to slightly biased flanking sequences or differences in mobility patterns of specific repeat units as previously mentioned by other investigators [12,13]. Nonetheless, this did not interfere with the overall results, as alleles were correctly assigned with or without adjustment of the number of repeats deduced by capillary electrophoresis.

The specificity of the present assay was demonstrated by the amplification of all 5 VNTRs in all Norwegian isolates. As the primers were designed to exclusively detect *F. noatunensis* subsp. *noatunensis* isolates from cod, the lack of amplification from some loci in other bacterial subspecies/species may be due to either sequence variability in the primer regions or the absence of the locus altogether.

Generally, informative VNTR loci require a good balance between variability and stability. Although the studied loci appeared relatively stable over several generations *in vitro*, a single repeat change was previously reported for fnVNTR-4 in *F. noatunensis* subsp. *noatunensis* GM2212 following successive culturing at 20°C [11]. Nevertheless, the fact that isolates from different outbreaks in the same locality (exemplified by MVT 10), displayed identical MLVA patterns over a period of 3 years, indicates that the loci are of sufficient stability to be used as VNTR markers for typing of *F. noatunensis* subsp. *noatunensis*.

Our MLVA assay provided high resolution typing and clustered the Norwegian *F. noatunensis* subsp. *noatunensis* isolates into three major clonal groups, consistent with previous MLVA findings [11]. However, in the previous study, they indicated low diversity among 17 *F. noatunensis* subsp. *noatunensis* isolates collected from outbreaks in cultured cod populations. A total of four different allelic profiles were identified compared with 13 MLVA profiles detected from the 91 outbreak isolates included in the present study. Although polymorphisms in fnVNTR-1 may have contributed to the greater discriminatory ability of the present assay, it is more likely related to the greater number of isolates included in the present analysis. It was also reported that isolates from wild cod are more diverse than those from farmed cod [11]. That the least polymorphic locus identified in the present study (fnVNTR-2) was among the most diverse loci identified in isolates from wild cod [11], suggests that the discriminatory power of the present assay for *F. noatunensis* subsp. *noatunensis* may be underestimated.

At the time of sampling, the epidemiological situation was complex with movement of juvenile fish common over long distances along the Norwegian coast. Thus, the information generated during the present study is difficult to interpret in terms of ‘natural’ or anthropological spread of infection. Our data shows, however, that several clones were present within the Norwegian cod-farming industry over a short period of time. Interpretation of these results in relation to microevolution will require knowledge of the mutation rates and understanding of how VNTRs in *F. noatunensis* subsp*. noatunensis* change with time. The situation could also be explained by the existence of many clones of which we have only identified a few. MVT1 may represent a highly successful spreading clone, as this genotype was detected at high frequency each sampling year. As with other theories relating to bacterial clonal expansion, the widespread dissemination of MVT1 could have been caused by human activities [11,14]. While any significance of the demonstrated diversity within *noatunensis* to pathogenesis remains unclear, the current study helps define and differentiate epizootic *F. noatunensis* clones causing francisellosis in Norwegian cod populations.

Our data indicated better resolution for MLVA compared to PFGE. The two methods, however, assess different genetic events in different parts of the chromosome. While mutation, recombination or replication error may affect the number and frequency of tandem repeats, variations in PFGE profiles are caused by mutations in restriction enzyme sites. Generally, isolates belonging to the same cluster or PFGE type were identified with the same MLVA type. It should be noted, however, that isolates used for PFGE analysis were chosen based on differences in MLVA profile, which might have introduced selection bias. We observed two isolates, including the type strain NCIMB14265^T^ that were inseparable by MLVA, but were resolved by PFGE. The PFGE profiles of these isolates differed by only one band, consistent with a single genetic event such as a point mutation, insertion or deletion [15]. NCIMB14265^T^ has been cultured repeatedly over time, which could explain the minor genetic changes.

## Conclusions

We found the described MLVA scheme to meet all the performance criteria proposed for a good typing method [16]. It shows good stability, provided 100% typeability of the isolates, is reproducible and has a high discriminatory power. These performance qualities do not differ significantly from the previous *F. noatunensis* MLVA assay [11], however, the main advantage of our MLVA is the high throughput that is facilitated by using a single multiplex PCR and capillary electrophoresis. The epidemiological concordance was demonstrated by the ability of MLVA typing to correctly cluster isolates within an outbreak and separate these from epidemiologically unrelated isolates. Thus, the assay has a great potential as a high-resolution molecular typing tool for the study of outbreaks of cod francisellosis. By using allele string codes based on repeat copy numbers for strain identification, the assay can easily be standardised to facilitate exchange of data among laboratories. Compared to PFGE, it lacks subjectivity, is less time-consuming and more discriminatory. However, as MLVA and PFGE target different parts of the chromosome, combined analyses of these two methods may result in a more discriminatory approach to understanding the processes of transmission of this fish pathogen in aquaculture.

## Methods

### Bacterial strains and culture conditions

Ninety-one isolates of *F. noatunensis* subsp. *noatunensis*, including the type strain NCIMB14265^T^, from disease outbreaks in Norwegian cod farms during the period 2005 to 2011, were studied. For comparative purposes, single isolates derived originally from Atlantic salmon *Salmo salar* L. (*F. noatunensis* subsp. *noatunensis* PQ 1106) in Chile, three line-grunt *Parapristipoma trilineatum* Thunberg (*F. noatunensis* subsp. *orientalis* Ehime-1) in Japan, and from a captive wild Atlantic cod (*F. noatunensis* subsp. *noatunensis*) in Ireland were included in the analysis. The collection also included three reference strains of *F. philomiragia* (CCUG 12603, CCUG 13404 and CCUG 19701). Details of source of isolation and country of origin of the strains can be found in supplementary material Additional file 1: Figure S1. Stock cultures of bacterial strains were stored in cryo-broth with 20% (v/v) glycerol at -80°C. Individual colonies of each strain were revived by streaking on Cysteine Heart Agar with Blood (CHAB) and incubated at 22°C for 7 days. *F. philomiragia* was incubated under the same conditions for 3 days. The Irish isolate was kindly provided by Dr Neil Ruane, Marine Institute, Oranmore, Ireland. The Chilean PQ 1106 isolate and the two *F. noatunensis* subsp. *orientalis* strains were kindly provided by PHARMAQ AS, Norway.

### Identification and selection of VNTR loci

Tandem repeats (TRs) were identified in the published complete genome sequence of *F. philomiragia* subsp*. philomiragia* (ATCC 25017) and three draft genome sequences of *F. noatunensis* subsp. *noatunensis* (NCIMB14265^T^, GM 2212^T^, PQ 1106) [17], using Tandem Repeats Finder (http://tandem.bu.edu/trf/trf.html) [18]. Initially, variability of 25 potential VNTR loci were tested in temporally and geographically discrete *F. noatunensis* subsp. *noatunensis* isolates using conventional PCR and gel-based analysis. Primers targeting flanking regions of each locus were designed using Primer3 software [19]. DNA was prepared by boiling bacterial cells in TZ lysis buffer [20] and used directly in PCR after brief centrifugation at 10,000 × g for 3 minutes. Alternatively, genomic DNA was purified using the QIAamp DNA mini kit (Qiagen) following the manufacturer’s instructions. PCR amplification reactions were carried out with a PTC- 100 Programmable Thermal Controller (MJ Research Inc. Watertown, Massachusetts, USA). Each 25-μl reaction mixture contained 1 × PCR buffer, 0.4 μM of each primer (Invitrogen), 0.2 mM dNTP mix, 1.5 mM MgCl_2_, 2U Taq DNA polymerase (GE Healthcare) and 2 μl of DNA as template. The thermal cycling conditions were as follows: Initial denaturation cycle at 95°C for 5 min, followed by 35 cycles of amplification at 95°C for 30 s, 53°C for 30 s and 72°C for 1 min and a final extension at 72°C for 4 min. A portion of the amplified PCR products (10 μl) were size-fractionated by electrophoresis on 3% agarose gel in 1 × TBE buffer at constant voltage of 70 V (3–4 V/cm) for 4 h and visualized after staining with ethidium bromide. As control, DNA from NCIMB14265^T^ was included in each experiment. Loci resulting in polymorphic banding patterns were considered suitable for further analysis.

### MLVA typing

Five VNTR loci were selected for MLVA and multiplexed in a single PCR reaction. Forward primers were labelled at the 5′ end with either VIC, NED or 6-carboxyfluorescein (6-FAM) fluorescent reporter dyes (Applied Biosystems). Reverse primers were unlabeled (Invitrogen). All primer sequences are shown in Table 2. PCRs were performed using the Qiagen multiplex PCR kit (Qiagen) in a 25 μl reaction following the manufacturer’s instructions. The optimum primer concentrations are shown in Table 2. The thermal cycling conditions were as follows: 15 min initial denaturation at 95°C, 30 cycles of amplification (30 s at 94°C, 90 s at 57°C and 90 s at 72°C), with a final extension of 30 min at 60°C. Arbitrarily chosen PCR products were verified by gel electrophoresis prior to fragment analysis using capillary electrophoresis. The PCR products were diluted 1:67 in deionized water and 1 μL of the diluted sample was added to 12 μL formamide and 1 μL of Geneflo-625 TAMRA internal size standard (CHIMERx, Madison, WI, USA). All samples were denaturated for 2 min at 94°C and cooled to room temperature before being subjected to capillary electrophoresis on a 3130*xl* Genetic Analyzer (Applied-Biosystems). Samples were run for 35 min at 60°C with an injection voltage of 15 kV for 5 s and a running voltage of 15 kV using POP-7 polymer (Applied-Biosystems). Each VNTR locus was identified by colour and size using GeneMapper software® 3.7 (Applied-Biosystems). Allele variation was identified by differences in fragment size. Fragment sizes were converted into repeat numbers based on the formula: number of repeats (bp) = fragment size (bp) − flanking regions (bp)/repeat size (bp). Flanking regions were deduced from the sequenced PCR products of several strains. Each isolate was assigned an MLVA type (MVT) based on a 5-digit allele string in the order fnVNTR-1, fnVNTR-2, fnVNTR-3, fnVNTR-4 and fnVNTR-5, reflecting the number of repeats at each locus. Absent PCR-products were designated an allele number of ‘0’. The allele strings were entered into BioNumerics software v6.1 (Applied Maths) as character values for clustering analysis.

**Table 2:** Primers used for MLVA in this study

**Marker**	**Primer sequence**^**a**^		**Primer concentration**
	**Forward**	**Reverse**	
fnVNTR-1	**V-**CAGCTAGAAGCTTATTCGCCTCTT	CAGGGTAATGCCTTAACGCATAT	0.2 μM
fnVNTR-2	**F-**GAGTATTCCCTGCACCTACAATGAT	TGTCATGCCTTTTTCTCTAGAGGAT	0.2 μM
fnVNTR-3	**N-**CAAACTCTTTCACAAGAGGAAGCAT	TCTGAACTCTGCTCTTTTCCCTCTA	0.2 μM
fnVNTR-4	**F-**TTGTGCAAACACATCGATAGGAGAT	AACTGCATCATCAGCATCTCTTCTA	0.2 μM
fnVNTR-4^b^	GTGCTTTTGCCTGTACCACCTT	GCATCATTTGATACACCGTCCA	NA
fnVNTR-5	**N-**CAATCACTCATCAACCACTAGCCAT	TGCTGACTAATGGCTGACTGTAGTT	0.3 μM
fnVNTR-5^b^	CTCATCAACCACTAGCCATCACAT	AAAGCCTGGGCTATCTAAATGCT	NA

^a^Fluorescent dyes are abbreviated as follows: F, FAM (6-carboxyfluorescein); N, NED; V, VIC. ^b^sequencing primers; NA, not applicable.

### Verification by DNA sequencing

To verify the accuracy of sizing determined by capillary electrophoresis, PCR fragments representing all variants of each tandem repeat were sequenced. For fnVNTR-4 and fnVNTR-5 new sequencing primers were designed (Table 2), while for the other loci the primers used to amplify the VNTRs were also used for sequencing. PCR products were purified using Nucleospin® Extract II (Macherey-Nagel, Germany) following the manufacturer’s instructions. Two μL of purified PCR product was added to 4 μL DYEnamic™ Dye Terminator Cycle Sequencing Kit (Amersham Biosciences), 1 μL sequencing primer (5 μM) and 3 μl sterile dH_2_O. Sequencing products were purified using the DYE Terminator Removal Kit (ABgene®) and sequenced on MegaBACE 1000 sequencing instrument (Amersham Biosciences). Consensus sequences were determined using Vector NTI (Invitrogen).

### Stability of VNTR loci after *in-vitro* passage

The stability of each VNTR locus was determined *in-vitro* after a number of laboratory passages (40 passages at 5 days intervals) using four *F. noatunensis* subsp. *noatunensis* isolates showing distinct MLVA profiles. For each passage, single colonies were streaked on CHAB agar plates. Bacterial growth conditions, preparation of DNA, VNTR PCR amplification and MLVA were carried out on single colonies from passage 10, 20, 30 and 40 as described above.

### PFGE typing

Prior to PFGE analysis, *in silico* searches for rare-cutting restriction enzymes were made from the available in-house *Francisella* genomes [17]. In all, five candidate endonucleases (*Pas*I, *Sac*II, *Apa*I, *Nae*I) were evaluated. Of these enzymes, *Pas*I (Fermentas) was the most discriminatory on the preliminary panel of five *F. noatunensis* isolates tested (data not shown). PFGE using *Pas*I was then performed on a subset of 54 isolates including members from each representative MLVA group, using the PulseNet protocol for subtyping of *Francisella tularensis* (CDC, Atlanta, GA, USA).

Bacterial cells were embedded in 1.2% agarose plugs (SeaKem Gold agarose; FMC Bioproduct, Rockland, ME), lysed, washed, and genomic DNA digested with 30 U *Pas*I enzyme for at least 3 h at 37°C. *Salmonella enterica* serotype Braenderup (H9812) was used as a reference standard, and restricted with 50 U *Xba*I (Roche Diagnostics, Indianapolis, IN, USA) for 3 hours at 37°C. The digests were resolved by electrophoresis on the CHEF-DRIII apparatus (Bio-Rad Laboratories, Hercules, CA) with 1% agarose gel in 0.5× Tris-borate –EDTA (TBE) buffer and running conditions set at voltage, 6 V/cm; initial switch time, 5.0 s; final switch time 30 s; runtime 18 h at 14°C. Gels were stained with ethidium bromide (1 mg/mL) and images of individual DNA fingerprints were captured using the Bio-Rad Gel Doc system (Bio-Rad Laboratories).

### Data analysis

Cluster analysis of MLVA and PFGE profiles was performed in BioNumerics 6.1 (Applied Maths, Saint-Martens-Latem, Belgium). Dendrograms were created using the categorical similarity coefficient or the Dice correlation coefficient coupled with WARD algorithm. A minimum spanning tree (MST) was created to illustrate the distribution and inter-relationships of the MLVA genotypes within the *F. noatunensis* subsp. *noatunensis* population (Figure 2). The discriminatory capacity of the MLVA assay was evaluated using the Simpson’s index of diversity [21], which was calculated for both individual and combined VNTR markers on a data subset containing single representatives of each genotype identified in individual diagnostic cases. The degree of linkage disequilibrium, as an indicator of the statistical independence of each of the five loci was tested using the LIAN Linkage analysis 3.5 Software as described by Haubold & Hudson [22] on a data subset representing single representatives of each genotype.

## Competing interests

The authors declare that they have no competing interests.

## Authors’ contributions

DS was a major contributor to project design, performed some of the experiments and responsible for writing of the manuscript. WX identified the VNTR loci in the draft genome, performed some of the experiments and contributed to writing of the manuscript. TNM performed and optimized the MLVA assay. LP, MK, SA identified the VNTR and restriction enzyme markers and contributed to completion of the manuscript. MF was involved in the interpretation of the data and contributed to the completion of the manuscript. CDJ conceived and coordinated the study, and contributed to completion of the manuscript. All authors read and approved the final manuscript.

## Supplementary Material

Additional file 1: Figure S1 Description of the *Francisella* isolates and strains used in the present study. The dendrogram was generated based on their MLVA genetic relatedness using categorical coefficient and Ward algorithm. nd = no data. Click here for file
