# Supplementary material for: An improved multiple-locus variable-number of tandem repeat analysis (MLVA) for the fish pathogen Francisella noatunensis using capillary electrophoresis
Source: BMC Vet Res. 2013 Dec 13;9:252. doi: 10.1186/1746-6148-9-252 (PMC3878797; doi:10.1186/1746-6148-9-252)
Supplement: Additional file 1: Figure S1 — Description of the Francisella isolates and strains used in the present study. The dendrogram was generated based on their MLVA genetic relatedness using categorical coefficient and Ward algorithm. nd = no data. [file 1746-6148-9-252-S1.pdf]

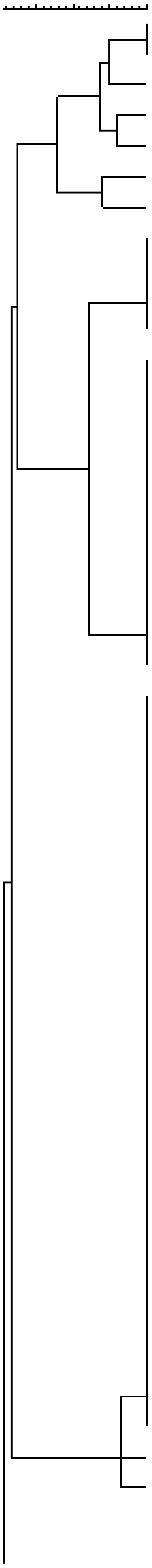

Strain/isolate	Year	Host/source	Province/county	Country	Allele-string	MLVA type	PFGE type
5409	2006	Tilapia	Costa Rica	Costa Rica	23-0-0-0-0	16	H
5887	2001	Three-lined grunt	Uwajima, Ehime prefecture	Japan	23-8-10-0-0	16	H
CCUG13.	1979	Human	Zurich	Switzerland	32-8-0-0-0	20	K
CCUG12.	1959	moribund muskrat	Brigham City, Utah	USA	28-0-4-0-0	19	J
CCUG19.	1960	Water	Utah	USA	25-0-10-0-0	18	I
5888	2006	Atlantic salmon	Lake Llanquihue	Chile	23-32-10-0-15	14	F
7061	2009	Atlantic cod	Waterford	Ireland	28-32-10-5-15	15	G
6062	2008	Atlantic cod	Møre og Romsdal	Norway	20-14-7-4-31	11	
6063	2008	Atlantic cod	Møre og Romsdal	Norway	20-14-7-4-31	11	D
6213	2008	Atlantic cod	Møre og Romsdal	Norway	20-14-7-4-31	11	
6214	2008	Atlantic cod	Møre og Romsdal	Norway	20-14-7-4-31	11	D
5590	2006	Atlantic cod	Møre og Romsdal	Norway	20-14-7-4-30	10	
5591	2006	Atlantic cod	Møre og Romsdal	Norway	20-14-7-4-30	10	
5592	2006	Atlantic cod	Møre og Romsdal	Norway	20-14-7-4-30	10	D
5593	2006	Atlantic cod	Møre og Romsdal	Norway	20-14-7-4-30	10	D
5594	2006	Atlantic cod	Møre og Romsdal	Norway	20-14-7-4-30	10	D
5949	2007	Atlantic cod	Møre og Romsdal	Norway	20-14-7-4-30	10	D
5950	2007	Atlantic cod	Møre og Romsdal	Norway	20-14-7-4-30	10	
6009	2007	Atlantic cod	Møre og Romsdal	Norway	20-14-7-4-30	10	
6065	2008	Atlantic cod	Møre og Romsdal	Norway	20-14-7-4-30	10	D
6154	2008	Atlantic cod	Møre og Romsdal	Norway	20-14-7-4-30	10	
6215	2008	Atlantic cod	Møre og Romsdal	Norway	20-14-7-4-30	10	
5331	2005	Atlantic cod	Hordaland	Norway	31-14-8-7-15	9	
5509	2006	Atlantic cod	Vl-Bergen	Norway	31-14-8-7-15	9	
5511	2006	Atlantic cod	Vl-Bergen	Norway	31-14-8-7-15	9	
5518	2006	Atlantic cod	Southern Skagerrak	Sweden	31-14-8-7-15	9	
5953	2007	Atlantic cod	Troms	Norway	31-14-8-7-15	9	
5954	2007	Atlantic cod	Troms	Norway	31-14-8-7-15	9	
5955	2007	Atlantic cod	Sogn og Fjordane	Norway	31-14-8-7-15	9	B
6278	2008	Atlantic cod	Tromsø Univ	Norway	31-14-8-7-15	9	
6280	2008	Atlantic cod	Tromsø Univ	Norway	31-14-8-7-15	9	
6281	2008	Atlantic cod	Troms	Norway	31-14-8-7-15	9	
6567	2008	Atlantic cod	Sogn og Fjordane	Norway	31-14-8-7-15	9	B
6568	2008	Atlantic cod	Sogn og Fjordane	Norway	31-14-8-7-15	9	
6569	2008	Atlantic cod	Sogn og Fjordane	Norway	31-14-8-7-15	9	B
6570	2008	Atlantic cod	Sogn og Fjordane	Norway	31-14-8-7-15	9	B
6571	2008	Atlantic cod	Sogn og Fjordane	Norway	31-14-8-7-15	9	B
6573	2008	Atlantic cod	Sogn og Fjordane	Norway	31-14-8-7-15	9	B
6574	2008	Atlantic cod	Sogn og Fjordane	Norway	31-14-8-7-15	9	B
6575	2008	Atlantic cod	Sogn og Fjordane	Norway	31-14-8-7-15	9	
6576	2008	Atlantic cod	Sogn og Fjordane	Norway	31-14-8-7-15	9	B
7119	2009	Atlantic cod	Vl-Bergen	Norway	31-14-8-7-15	9	B
7123	2007	Atlantic cod	Sogn og Fjordane	Norway	31-14-8-7-15	9	B
7125	2007	Atlantic cod	Sogn og Fjordane	Norway	31-14-8-7-15	9	B
7128	2008	Atlantic cod	Sogn og Fjordane	Norway	31-14-8-7-15	9	
7129	2008	Atlantic cod	Sogn og Fjordane	Norway	31-14-8-7-15	9	
NCIMB1.	2005	Atlantic cod	Hordaland	Norway	31-14-8-7-15	9	C
6572	2008	Atlantic cod	Sogn og Fjordane	Norway	32-14-8-7-15	12	B
6577	2008	Atlantic cod	Sogn og Fjordane	Norway	31-14-9-7-15	13	B

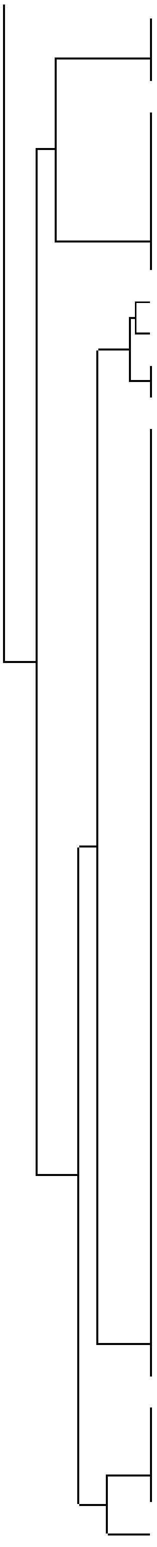

6472	2008	Atlantic cod	Møre og Romsdal	Norway	33-8-11-4-10	5	
6496	2008	Atlantic cod	nd	Norway	33-8-11-4-10	5	A
7127	2008	Atlantic cod	Rogaland	Norway	33-8-11-4-10	5	A
5358	2005	Atlantic cod	Rogaland	Norway	32-8-9-6-10	6	A
5359	2005	Atlantic cod	Rogaland	Norway	32-8-9-6-10	6	
5360	2005	Atlantic cod	Rogaland	Norway	32-8-9-6-10	6	A
6471	2008	Atlantic cod	Møre og Romsdal	Norway	32-8-9-6-10	6	
6476	2008	Atlantic cod	Nordland	Norway	32-8-9-6-10	6	A
7126	2008	Atlantic cod	Rogaland	Norway	32-8-9-6-10	6	
5396	2006	Atlantic cod	Rogaland	Norway	29-8-10-6-10	8	A
6684	2009	Atlantic cod	Nordland	Norway	31-8-10-6-10	4	A
8075	2011	Atlantic cod	Møre og Romsdal	Norway	27-8-10-6-10	7	A
8087	2011	Atlantic cod	Møre og Romsdal	Norway	27-8-10-6-10	7	
5336	2005	Atlantic cod	Pharmac AS	Norway	30-8-10-6-10	1	
5339	2005	Atlantic cod	Sogn og Fjordane	Norway	30-8-10-6-10	1	A
5340	2005	Atlantic cod	Sogn og Fjordane	Norway	30-8-10-6-10	1	A
5341	2005	Atlantic cod	Sogn og Fjordane	Norway	30-8-10-6-10	1	E
5347	2005	Atlantic cod	Pharmaq AS	Norway	30-8-10-6-10	1	
5348	2005	Atlantic cod	Pharmaq AS	Norway	30-8-10-6-10	1	
5350	2005	Atlantic cod	Pharmaq AS	Norway	30-8-10-6-10	1	A
5393	2006	Atlantic cod	Rogaland	Norway	30-8-10-6-10	1	A
5394	2006	Atlantic cod	Rogaland	Norway	30-8-10-6-10	1	
5395	2006	Atlantic cod	Rogaland	Norway	30-8-10-6-10	1	A
5397	2006	Atlantic cod	Rogaland	Norway	30-8-10-6-10	1	
5413	2006	Atlantic cod	Sogn og Fjordane	Norway	30-8-10-6-10	1	
6022	2007	Atlantic cod	Nordland	Norway	30-8-10-6-10	1	
6023	2007	Atlantic cod	Nordland	Norway	30-8-10-6-10	1	
6024	2007	Atlantic cod	Nordland	Norway	30-8-10-6-10	1	
6087	2008	Atlantic cod	Sogn og Fjordane	Norway	30-8-10-6-10	1	A
6088	2008	Atlantic cod	Sogn og Fjordane	Norway	30-8-10-6-10	1	A
6089	2008	Atlantic cod	Sogn og Fjordane	Norway	30-8-10-6-10	1	A
6090	2008	Atlantic cod	Sogn og Fjordane	Norway	30-8-10-6-10	1	A
6091	2008	Atlantic cod	Sogn og Fjordane	Norway	30-8-10-6-10	1	A
6337	2008	Atlantic cod	nd	Norway	30-8-10-6-10	1	
6420	2008	Atlantic cod	Møre og Romsdal	Norway	30-8-10-6-10	1	A
6682	2009	Atlantic cod	Nordland	Norway	30-8-10-6-10	1	A
6683	2009	Atlantic cod	Nordland	Norway	30-8-10-6-10	1	A
6685	2009	Atlantic cod	Nordland	Norway	30-8-10-6-10	1	
6686	2009	Atlantic cod	Nordland	Norway	30-8-10-6-10	1	A
6689	2009	Atlantic cod	VESO/VIKAN	Norway	30-8-10-6-10	1	
7621	2010	Atlantic cod	Hagarsneset	Norway	30-8-10-6-10	1	
7625	2010	Atlantic cod	Hagarsneset	Norway	30-8-10-6-10	1	
7667	2010	Atlantic cod	Møre og Romsdal	Norway	30-8-10-6-10	1	
7670	2010	Atlantic cod	Møre og Romsdal	Norway	30-8-10-6-10	1	
5349	2005	Atlantic cod	Pharmaq AS	Norway	30-8-10-6-11	2	
6086	2008	Atlantic cod	Sogn og Fjordane	Norway	30-8-10-6-11	2	A
6478	2008	Atlantic cod	Sogn og Fjordane	Norway	30-8-10-6-11	2	A
6479	2008	Atlantic cod	Sogn og Fjordane	Norway	30-8-10-6-11	2	
6422	2008	Atlantic cod	Møre og Romsdal	Norway	33-10-11-4-9	3	A
